# Inflammation-Induced Coagulopathy Substantially Differs Between COVID-19 and Septic Shock: A Prospective Observational Study

**DOI:** 10.3389/fmed.2021.780750

**Published:** 2022-01-17

**Authors:** Mélanie Dechamps, Julien De Poortere, Manon Martin, Laurent Gatto, Aurélie Daumerie, Caroline Bouzin, Marie Octave, Audrey Ginion, Valentine Robaux, Laurence Pirotton, Julie Bodart, Ludovic Gerard, Virginie Montiel, Alessandro Campion, Damien Gruson, Marie-Astrid Van Dievoet, Jonathan Douxfils, Hélène Haguet, Laure Morimont, Marc Derive, Lucie Jolly, Luc Bertrand, Laure Dumoutier, Diego Castanares-Zapatero, Pierre-François Laterre, Sandrine Horman, Christophe Beauloye

**Affiliations:** ^1^Pôle de Recherche Cardiovasculaire, Institut de Recherche Expérimentale et Clinique, Université Catholique de Louvain, Brussels, Belgium; ^2^Department of Cardiovascular Intensive Care, Cliniques Universitaires Saint-Luc, Brussels, Belgium; ^3^Computational Biology and Bioinformatics Unit, de Duve Institute, Université Catholique de Louvain, Brussels, Belgium; ^4^IREC Imaging Platform, Institut de Recherche Expérimentale et Clinique, Université Catholique de Louvain, Brussels, Belgium; ^5^Department of Intensive Care, Cliniques Universitaires Saint-Luc, Brussels, Belgium; ^6^Pôle de Pneumologie, Institut de Recherche Expérimentale et Clinique, Université Catholique de Louvain, Brussels, Belgium; ^7^Department of Clinical Biology, Cliniques Universitaires Saint-Luc, Brussels, Belgium; ^8^Department of Pharmacy, Namur Research Institute for Life Sciences, Namur, Belgium; ^9^Qualiblood, s.a., Namur, Belgium; ^10^Inotrem s.a., Vandoeuvre-les-Nancy, France; ^11^Experimental Medicine Unit, de Duve Institute, Université Catholique de Louvain, Brussels, Belgium; ^12^Division of Cardiology, Cliniques Universitaires Saint-Luc, Brussels, Belgium

**Keywords:** COVID-19, septic shock, inflammation, coagulopathy, platelet, NETosis, endothelium

## Abstract

Critical COVID-19, like septic shock, is related to a dysregulated systemic inflammatory reaction and is associated with a high incidence of thrombosis and microthrombosis. Improving the understanding of the underlying pathophysiology of critical COVID-19 could help in finding new therapeutic targets already explored in the treatment of septic shock. The current study prospectively compared 48 patients with septic shock and 22 patients with critical COVID-19 regarding their clinical characteristics and outcomes, as well as key plasmatic soluble biomarkers of inflammation, coagulation, endothelial activation, platelet activation, and NETosis. Forty-eight patients with matched age, gender, and co-morbidities were used as controls. Critical COVID-19 patients exhibited less organ failure but a prolonged ICU length-of-stay due to a prolonged respiratory failure. Inflammatory reaction of critical COVID-19 was distinguished by very high levels of interleukin (IL)-1β and T lymphocyte activation (including IL-7 and CD40L), whereas septic shock displays higher levels of IL-6, IL-8, and a more significant elevation of myeloid response biomarkers, including Triggering Receptor Expressed on Myeloid cells-1 (TREM-1) and IL-1ra. Subsequent inflammation-induced coagulopathy of COVID-19 also differed from sepsis-induced coagulopathy (SIC) and was characterized by a marked increase in soluble tissue factor (TF) but less platelets, antithrombin, and fibrinogen consumption, and less fibrinolysis alteration. In conclusion, COVID-19 inflammation-induced coagulopathy substantially differs from SIC. Modulating TF release and activity should be evaluated in critical COVID-19 patients.

## Introduction

The coronavirus disease 2019 (COVID-19) varies from asymptomatic to a severe form with acute respiratory distress syndrome (ARDS) and the pathophysiology of the severe form has yet to be fully elucidated. The variability of host immune responses suggests that dysregulated systemic inflammation, classically called a “cytokine storm,” could contribute to the pathogenesis of severe cases (1, 2). In addition, COVID-19 is associated with a high incidence of arterial and deep venous thrombosis leading to pulmonary embolism (3, 4) and pulmonary microthrombosis (5).

Several mechanisms underlying the pathophysiology of COVID-19 have been proposed. First, ribonucleic acid (RNA) viruses like SARS-CoV-2 usually initiate innate immunity through their detection by pattern recognition receptors (PRRs), triggering subsequent inflammatory and immune responses via the secretion of various cytokines (6). Supporting this hypothesis, critical COVID-19 has been associated with high levels of interferon-γ (IFN-γ), tumor necrosis factor-α (TNF-α), numerous interleukins (ILs), macrophage inflammatory proteins (MIPs), and IFN γ-induced protein 10 (IP-10) (1, 7, 8). Second, internalization of SARS-CoV-2 within endothelial cells after binding to angiotensin-converting enzyme 2 (ACE2) contributes to endotheliitis (9). Both direct injury and activation of the endothelium by dysregulated inflammation may lead to loss of endothelial barrier integrity and to the occurrence of a prothrombotic phenotype (10). Indeed, plasmatic level of vascular cell adhesion molecule-1 (VCAM-1), intercellular adhesion molecule 1 (ICAM-1) (11), plasminogen activator inhibitor-1 (PAI-1) (12), tissue factor (TF) (13), and von Willebrand factor (vWF) (4, 14) antigens and activities are all elevated in severe COVID-19 cases. However, a simultaneous increase in tissue plasminogen activator (tPA) or tissue factor pathway inhibitor (TFPI) could mitigate thrombosis formation (15).

Critical COVID-19-associated coagulopathy is characterized by high D-dimers due to increased thrombin generation and increased fibrinolysis (14, 16). In addition, platelets are hyperactivated (8, 17) and cooperate with the endothelium and immune cells to promote pulmonary microthrombosis through neutrophil extracellular trap (NET) formation. Dysregulated NETosis contributes to hypercoagulability and thrombosis (18). Accordingly, NET-containing microthrombi have been detected in COVID-19 pulmonary autopsies, and increased NET formation correlates with COVID-19-related ARDS and disease severity (19). Moreover, the interaction of platelets with monocytes triggers TF expression in severe COVID-19 patients, further exacerbating the hypercoagulable state (20).

Interestingly, sepsis and septic shock are well-known models of cytokine storms and coagulopathy (21). Like infection by RNA viruses, sepsis is initiated by activation of the innate immune system through recognition of pathogen-associated molecular patterns and damage-associated molecular patterns by PRRs. This inflammatory reaction enhances endothelial dysfunction and contributes to the procoagulant state with microvascular thrombi formation and, eventually, organ damage (22). The procoagulant state of sepsis is further amplified by NETs (23). Sepsis-induced coagulopathy (SIC) occurs as a continuum, progressing to disseminated intravascular coagulopathy (DIC) if the underlying etiology of the sepsis is not resolved. The clinical characteristics of critical COVID-19 and septic shock patients are extensively discussed in the literature although the entities have never been systematically compared in a prospective clinical setting including control patients with matched age, gender, and co-morbidities (24–26).

The current study prospectively compared patients with septic shock and those with critical COVID-19 on admission to the ICU regarding their clinical characteristics and outcomes, as well as key plasmatic soluble biomarkers of inflammation, coagulation, endothelial activation, platelet activation, and NETosis. Here, we show both coagulopathy and inflammatory pattern substantially differ between patients with septic shock and critical COVID-19, highlighting the specific actors involved in their respective pathogenesis.

## Methods

### Aim, Design, and Setting of the Study

This study comparing clinical outcomes, inflammatory reaction, and coagulopathy between critical COVID-19 and septic shock was a monocenter, prospective, translational observational study. Adult patients were systematically included between February 1, 2019, and June 1, 2020. The ethics committee approved the study protocol, and all patients signed their informed consent (B403201938590, NCT04107402). Protocol amendment was done to include COVID-19 patients in the ongoing study. All authors had full access to primary clinical data.

### Population

Patients with critical COVID-19 were those admitted to the ICU for moderate or severe ARDS due to SARS-Cov-2 infection; they were included within 5 days of admission. Acute respiratory distress syndrome was diagnosed according to the Berlin definition (27), and SARS-Cov-2 infection was demonstrated by real-time reverse transcription PCR on nasopharyngeal swabs. Septic shock was defined according to the Sepsis-3 definition as sepsis with vasopressor therapy needed to elevate the mean arterial pressure ≥65 mmHg and lactate levels >2 mmol/L despite adequate fluid resuscitation of 30 ml/kg of intravenous crystalloid within 6 h (28). A similar protective ventilation strategy (including positive end expiratory pressure above 5 cmH_2_O, maximum tidal volume of 6 ml/kg, and maximal plateau pressure of 30 cmH_2_O) was applied in both COVID-19 and septic shock patients with ARDS. Prone positioning and inhaled nitric oxide were used for severe ARDS, and venovenous extracorporeal membrane oxygenation (VV-ECMO) for refractory hypoxemia despite optimal treatment (29). Venovenous extracorporeal membrane oxygenation was never used in septic shock patients. Patients with septic shock admitted to the ICU were included within 2 days of admission. Control patients with matched age, gender, and co-morbidities were recruited at a central laboratory consultation. Similar exclusion criteria were applied to all groups: therapeutic anticoagulation (oral or parenteral, including heparins, fondaparinux, vitamin K antagonists, and direct oral anticoagulants), recent (within <1 month) chemotherapy, active inflammatory disease, hemophilia and other coagulopathies, previous history of thrombocytopenia (<100,000 platelets/mm^3^), cirrhosis (Child–Pugh >A), recent (within <48 h) major surgery (behalf, for septic shock, for infection source control), cardiac arrest during ICU stay, and decision of care limitation. All septic and COVID-19 cases received thromboprophylaxis using low-molecular-weight heparin (LMWH; nadroparin 3,800 IU/days subcutaneously). Sampling was performed at least 6 h after LMWH injection. For the patients with COVID-19, patients on antibiotics for any suspected or confirmed bacterial coinfections were formally excluded.

### Clinical Outcomes

Patient baseline characteristics and clinical outcomes were compared. Patient prognosis was assessed using acute physiologic assessment and chronic health evaluation II (APACHE II) (30) and sequential organ failure assessment (SOFA) (31) scores. Moreover, disseminated intravascular coagulation (DIC) and SIC were diagnosed using the International Society of Thrombosis and Hemostasis scoring at inclusion (32, 33). Data were collected from central medical records, including biological datasets that were routinely performed in patients admitted in ICU such as platelet count, CRP (C-reactive protein) level, coagulation assessment, renal function, and liver enzymology. Clinical outcomes were assessed 30 days after ICU admission. Bleeding complications were assessed with Thrombolysis in Myocardial Infarction (TIMI) bleeding criteria, frequently used for cardiovascular trials (34). A major bleeding is defined by the following criteria: any intracranial bleeding, clinically overt signs of hemorrhage associated with a drop in hemoglobin of ≥5 g/dl or a ≥15% absolute decrease in haematocrit and fatal bleeding.

### Sampling

Blood samples were collected through the central venous catheter in all ICU patients and by venous puncture in the control group. Venous blood was collected using vacutainer tubes containing CPDA. After two centrifugation runs enabling platelet isolation, plasma was collected, apportioned into 1 ml aliquots and stored at −80°C until use.

### Measurement of Biomarkers

Soluble biomarkers of inflammation, coagulation, endothelial and platelet activation, and NETosis were measured using enzyme-linked immunosorbent assay (ELISA) or suspension array sandwich immunoassays according to regulatory requirements for commercially available research use only ELISA assays. Frozen platelet poor plasma was thawed at room temperature the day of the experiment. The details of each markers analyzed are listed in the Supplementary Table 1. Each analytical run was performed in duplicate. Methods respected their respective validated lower limit of quantification and upper limit of quantification. Cytokines and chemokines were measured using Bio-Plex Pro Human Cytokine 27-Plex Panel (27-Plex) and Bio-Plex Human ICAM-VCAM (hICAM-hVCAM) following the manufacturer's protocol.

### Lung Biopsies and Autopsies

Lung biopsies and autopsies were obtained from the biolibrary of Cliniques Universitaires Saint-Luc. A “Human Material Transfer Agreement for research purpose” was elaborated between CUSL and UCLouvain. Use of Residual Human Body Material was approved by the local Ethic committee (B403201938590). Control patients, patients with ARDS from septic shock, and COVID-19 were compared. COVID-19 specimens were lung autopsies obtained from patients who died from respiratory failure at intensive care unit (ICU). Septic shock samples were biopsies taken owing to ARDS due to bacterial pulmonary or extra-pulmonary infection. Control specimens were archived tissues from patients who underwent lung surgery. Details about the methods and analyses are provided in the Supplementary Material (Supplementary Methods).

### Statistical Analyses

The analyses were conducted using GraphPad Prism Version 9 (GraphPad Software, San Diego, California). Continuous variables were expressed as mean ± standard deviation (SD) and categorical variables were expressed as number and percentage. The data were subjected to the Kolmogorov–Smirnov normality test and Bartlett's test for homogeneity of variance. Log transformations were performed when appropriate. The categorical variables were analyzed using the Chi-squared test or Fisher's exact test, and the continuous variables using Tukey's ordinary one-way ANOVA or an unpaired Student's *t*-test, as appropriate. A log-rank test was applied to compare ICU length of stay and ventilation duration. All *p*-values were two-sided, and *p* < 0.05 was considered statistically significant.

A principal component analysis (PCA) was conducted in R (35), based on the patients with COVID-19 or septic shock and study outcomes. As an exploratory multivariate analysis, PCA provides a condensed overview of the main sources of variability in a dataset composed of individuals (here, the patients) with a large number of variables measured (here, the study outcomes). The core idea is to reduce data dimensionality by building new latent variables—called the principal components (PCs)—from the measured ones. The PCs, which are orthogonal between each other, capture the main sources of data variability in a decreasing manner (PC1 captures more variability than PC2, etc.). Each original variable contributes with a certain weight (called a loading) to the construction of these PCs. Thereafter, the individuals are projected onto these PCs, with these projections called the scores. From this graphical representation, one can inspect the data structure with respect to the main sources of variability. This technique enables to explore the variables that seek to recover the grouped structure of the patients from the measured variables that are segregating the patients and the relationship between the variables of interest. Prior to PCA, the data were standardized, then imputed with the missMDA package (36), so that imputed values would not impact the factorial analysis results.

## Results

### Baseline Characteristics and Clinical Outcomes of Critical COVID-19 and Septic Shock

**Figure 1:**
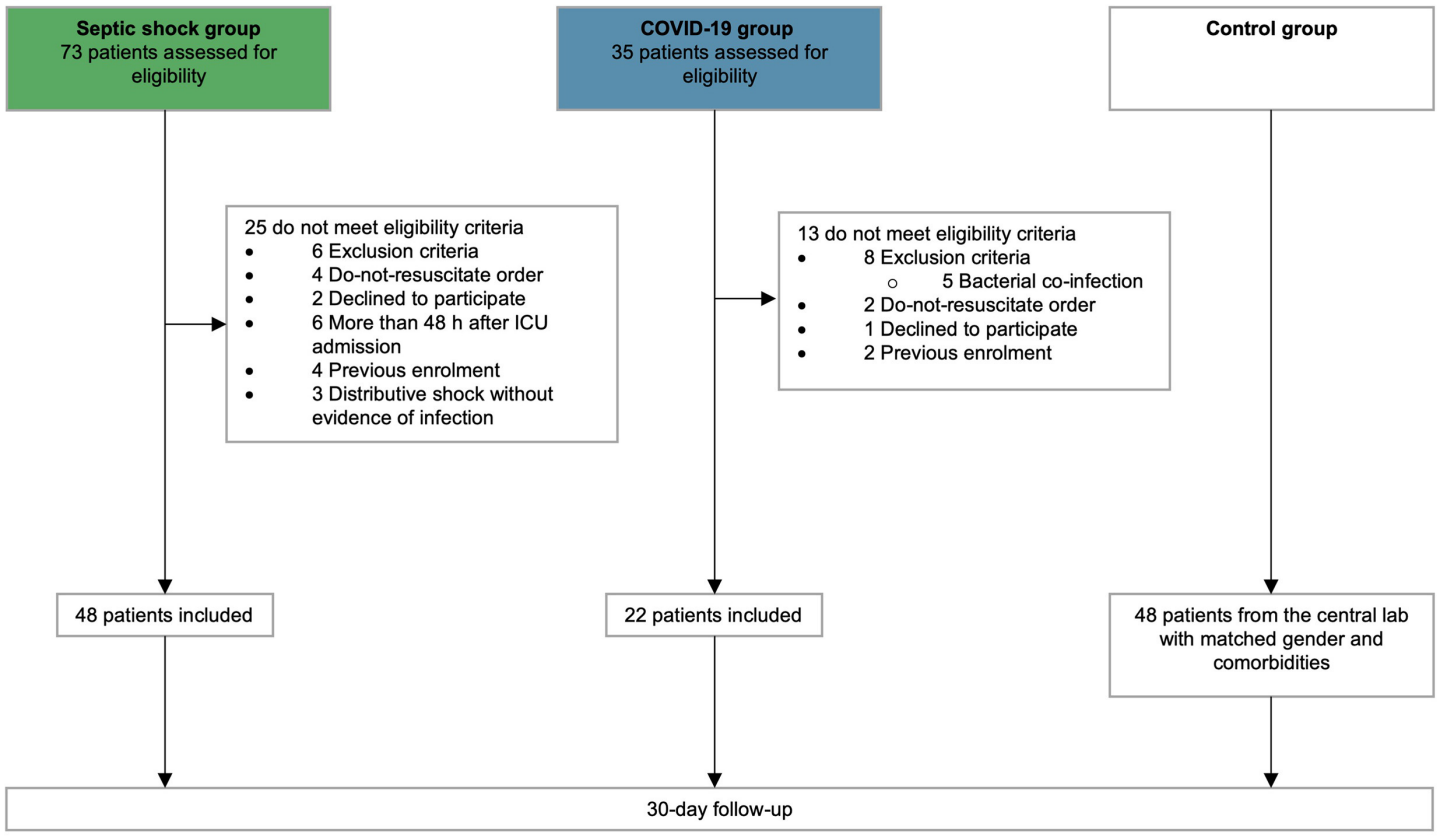
Flow chart. Flow chart of patients screening and inclusion.

Overall, 118 patients were enrolled, including 48 with septic shock, 22 with COVID-19, and 48 controls matched for age, gender, and main co-morbidities (flowchart, Figure 1). Type of infection and culture in septic shock patients are detailed in Supplementary Table 2. The baseline characteristics of all groups, as well as the clinical outcomes of septic shock and COVID-19 patients are detailed in Table 1.

**Table 1:** Baseline characteristics of prospective cohort of patients.

	**Control**	**COVID-19**	**Septic shock**	***p*-Value**
	***n* = 48**	***n* = 22**	***n* = 48**	
**Demographics**				
Men	26 (54)	15 (68)	24 (50)	0.36
Women	22 (46)	7 (32)	24 (50)	
Age (years)	61.9 ± 14.5	59.9 ± 10.3	65.0 ± 14.2	0.53
**Medical history**				
Hypertension	20 (42)	12 (56)	25 (52)	0.48
BMI >25	26 (58)	14 (74)	26 (54)	0.34
Diabetes	11 (23)	8 (36)	5 (10)	0.71
History of smoking	10 (21)	1 (5)	15 (31)	0.04
COPD	4 (8)	3 (14)	5 (10)	0.75
CKD	9 (19)	0 (0)	10 (21)	0.07
Cancer	15 (31)	0 (0)	9 (19)	0.01
**Delay symptoms-ICU admission (days)**		7.3 ± 3.2	2.6 ± 2.4	<0.01
**Routine laboratory testing**				
Highest CRP (mg/dl)		323 ± 119	313 ± 122	0.75
Creatinine (mg/dl)		0.91 ± 0.59	2.19 ± 1.91	<0.01
Hemoglobin (g/dl)		11.62 ± 1.90	10.34 ± 2.05	0.02
Neutrophils (10^3^/μl)		8.0 ± 3.0	15.5 ± 10.2	<0.01
Lymphocytes (/μl)		893 ± 319	779 ± 521	0.33
Lowest lymphocytes (10^3^/μl)		484 ± 335	469 ± 310	0.86
Platelet count (10^3^/μl)		271 ± 117	203 ± 143	0.04
**Organ failure, severity scores, and complications**				
PaO_2_/FiO_2_		103 ± 37	225 ± 119	<0.01
Mechanical ventilation		17 (77)	25 (52)	0.06
VV ECMO		5 (23)	0 (0)	<0.01
Norepinephrine (μg/kg/min)		0.049 ± 0.105	0.330 ± 0.350	<0.01
Norepinephrine duration (days)		1.2 ± 3.4	4.8 ± 6.1	<0.01
Renal replacement therapy		1 (5)	13 (27)	0.04
Apache II score		15 ± 4	20 ± 7	<0.01
SOFA score		4 ± 1	9 ± 3	<0.01
SIC		0 (0)	11 (24)	0.01
DIC		0 (0)	7 (16)	0.09
Thromboembolic events		6 (27)	4 (8)	0.06
TIMI major bleeding events		5 (23)	1 (2)	0.01
**Outcome**				
30-day mortality		6 (27)	22 (46)	0.45
Ventilation duration (days)		27 ± 24	4 ± 7	<0.01
ICU length of stay (days)		29 ± 30	8 ± 9	<0.01

*Values are numbers (percentages) or mean ± standard deviation. Major bleeding complications have been defined according to the TIMI definition. All bleeding complications in COVID-19 group occurred in ECMO-treated patients. APACHE, acute physiology and chronic health evaluation, BMI, body mass index; COPD, chronic obstructive pulmonary disease; CKD, chronic kidney disease; CRP, C-reactive protein; DIC, disseminated intravascular coagulopathy; ICU, intensive care unit; PaO_2_/FiO_2_, arterial oxygen partial pressure/fractional inspired oxygen; SIC, sepsis-induced coagulopathy; SOFA, sepsis-related organ failure assessment; VV ECMO, venovenous extracorporeal membrane oxygenation*.

The demographic characteristics and past medical history were similar among the three groups, except that the COVID-19 group included less smokers and oncologic patients. Before inclusion into the study, most COVID-19 patients had been treated with hydroxychloroquine (*n* = 18, 82%) but only one had received corticosteroids (methylprednisolone) as compared with five septic shock patients receiving low-dose hydrocortisone. At the end of the ICU stay, five COVID-19 patients (19%) and 22 septic shock patients (46%) had been treated with corticosteroids. The time delay between symptom onset and ICU admission was longer for COVID-19 compared with septic shock (2.6 ± 2.4 days and 7.3 ± 3.2 days, respectively, *p* < 0.01). The patients with septic shock displayed worse severity scores due to multiple organ failure. By contrast, the COVID-19 patients presented with more severe respiratory failure, as indicated by a lower PaO_2_/FiO_2_ (arterial oxygen partial pressure/fractional inspired oxygen) ratio, a higher rate of mechanically ventilated patients, longer ventilation duration and ICU length of stay. The 30-day mortality did not differ between both groups.

### Coagulopathies in Critical COVID-19 and Septic Shock Patients

**Figure 2:**
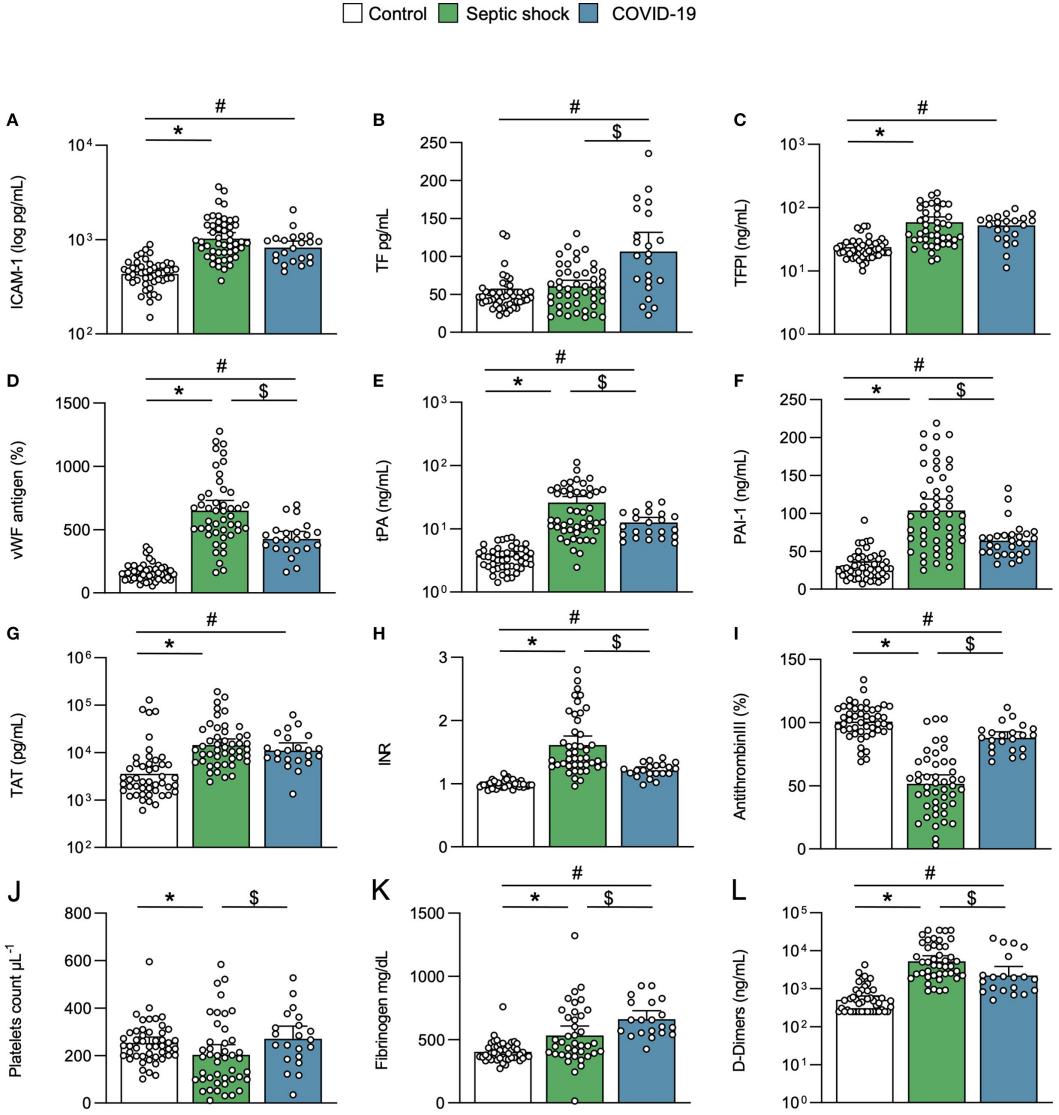
Endothelial activation and coagulation. Scatter graphs of soluble biomarkers of **(A–F)** endothelial activation and **(G–L)** coagulation. Individual values (open circle), mean (colored rectangle), and standard deviation are presented on the graphs. *p < 0.05 between septic shock and control patients. ^#^*p* < 0.05 between COVID-19 and control patients. ^$^*p* < 0.05 between septic shock and COVID-19 patients. ICAM-1, intercellular adhesion molecule-1; INR, international normalized ratio; PAI-1, plasminogen activator inhibitor-1; TAT, thrombin–antithrombin complex; TF, tissue factor; TFPI, tissue factor pathway inhibitor; tPA, tissue plasminogen activator; vWF, von Willebrand factor.

The critical COVID-19 and septic shock cases were compared with the matched controls considered as a reference. Circulating levels of ICAM-1, reflecting endothelial dysfunction, were similarly increased in both COVID-19 and septic shock patients, compared to matched controls (Figure 2A). Other endothelial biomarkers differed between COVID-19 and septic shock. Circulating TF was higher in COVID-19 patients than septic shock patients, while TFPI was similarly elevated in both groups (Figures 2B,C). By contrast, vWF, PAI-1, and tPA were predominant in septic shock (Figures 2D–F). The difference in TF levels did not impact thrombin generation, as reflected by the thrombin–antithrombin complex (TAT), which was similarly increased in critical COVID-19 and septic shock, compared to matched controls (Figure 2G). Both critical conditions led to increased international normalized ratio (INR) and antithrombin consumption, while these changes were more pronounced in septic shock (Figures 2H,I). Interestingly, platelet and fibrinogen consumption mainly occurred in septic shock (Figures 2J,K). Accordingly, SIC and DIC were diagnosed in 24 and 16% of septic shock cases, respectively, but in none of the critical COVID-19 cases (*p* < 0.05). D-dimers were significantly elevated in both COVID-19 and septic shock patients compared with matched controls, but further in septic shock compared with COVID-19 patients (Figure 2L). Of interest, the levels of TAT and D-dimers that were measured in several control patients with co-morbidities were like those found in some critically ill patients, whether they had COVID-19 or not.

### Platelet Activation and NETosis in Critical COVID-19 and Septic Shock Patients

**Figure 3:**
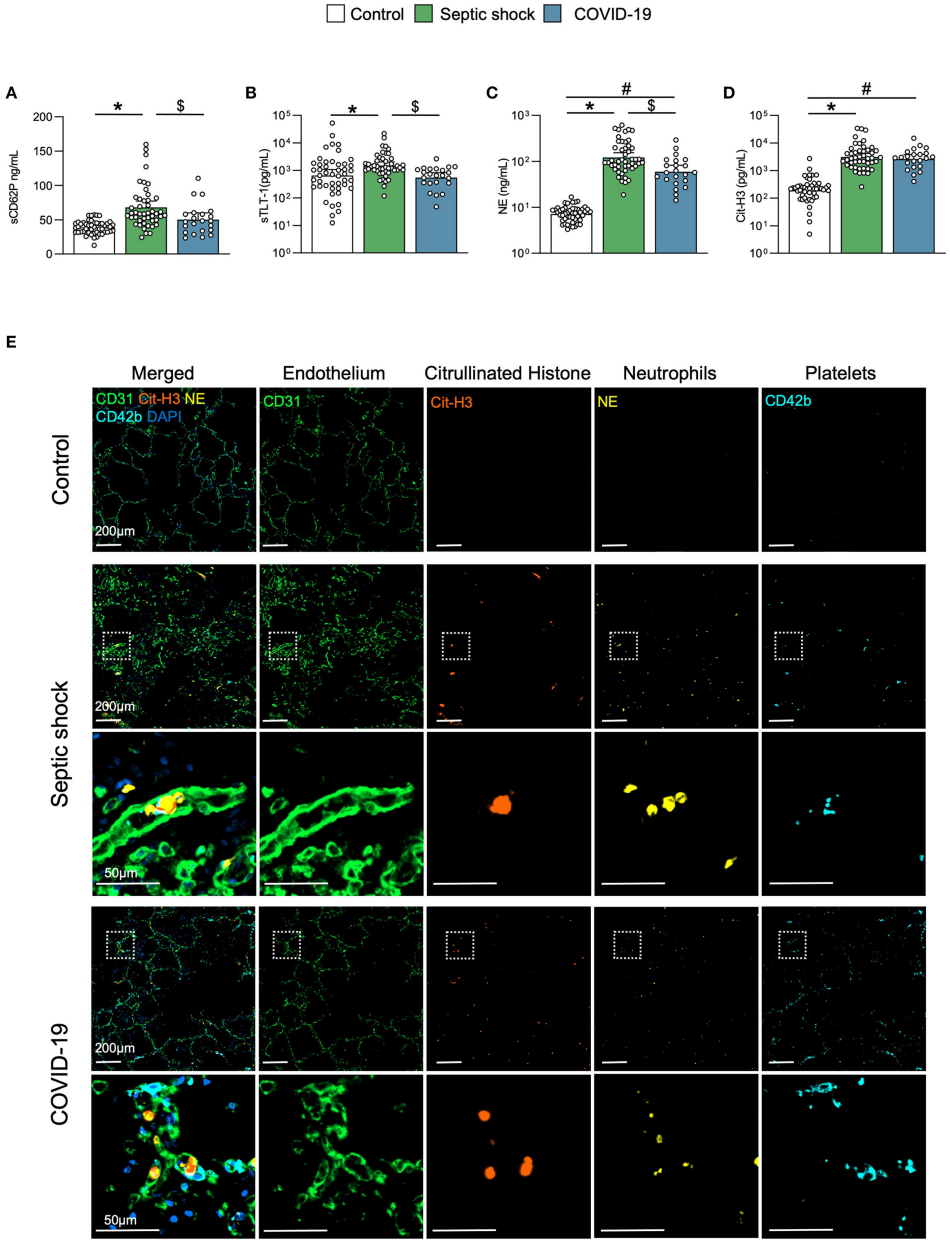
Platelet activation and NETosis. Scatter graphs of soluble biomarkers of **(A,B)** platelet activation and **(C,D)** NETosis. Individual values (open circle), mean (colored rectangle), and standard deviation are presented on the graphs. *p < 0.05 between septic shock and control patients. ^#^*p* < 0.05 between COVID-19 and control patients. ^$^*p* < 0.05 between septic shock and COVID-19 patients. **(E)** Representative microscopy pictures of endothelial cells (CD31, green), Cit-H3 (orange), neutrophils (NE, yellow), platelets (CD42b, light blue), nucleus (DAPI, dark blue), and merged staining, from control, septic shock, and COVID-19 lungs. sCD62P, soluble p-selectin; Cit-H3, citrullinated histone 3; NE, neutrophil elastase; NET, neutrophil extracellular traps; MPO, myeloperoxidase; sTLT-1, soluble triggering receptor expressed on myeloid cells (TREM) like transcript-1.

Platelet activation soluble biomarkers P-selectin (sCD62P) and triggering receptor expressed on myeloid cells (TREM)-like transcript-1 (sTLT-1) were significantly increased in septic shock, but not in COVID-19 (Figures 3A,B). Levels of circulating NE and Cit-H3, reflecting neutrophil activation and NETosis, respectively, were increased in both diseases compared to matched controls but there were no differences between septic shock and COVID-19 (Figures 3C,D). This result was supported by the histopathological analysis of lung sections from control patients and patients with critical COVID-19- and septic shock-induced ARDS (Figure 3E). The baseline characteristics of patients included in the histopathological analysis are described in the Supplementary Table 3. As already described (37–39), vascular immunothrombosis containing platelets and neutrophils was observed in the lungs of patients with COVID-19 and septic shock, but not in the controls.

### Inflammation and Immune Response in Critical COVID-19 and Septic Shock Patients

**Figure 4:**
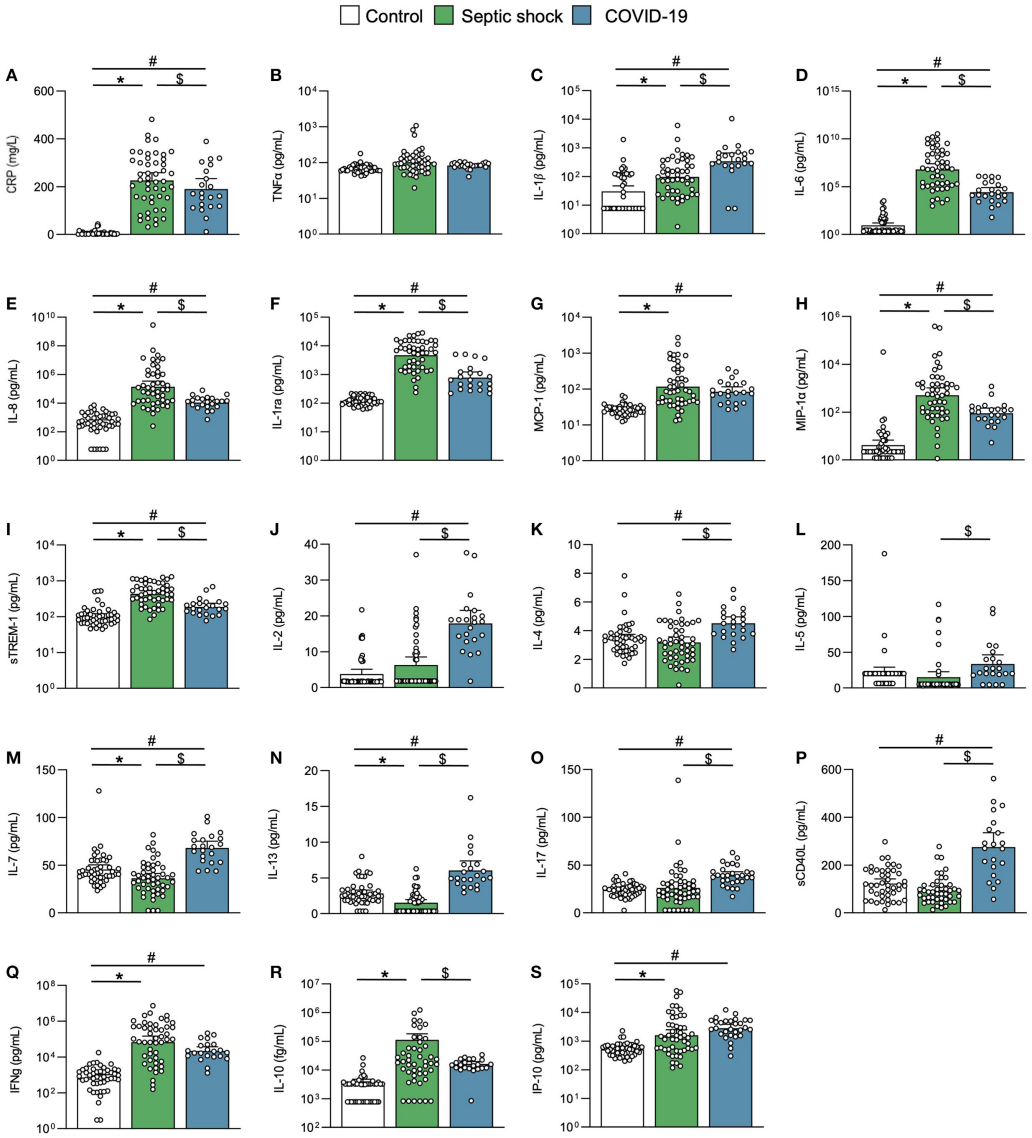
Inflammation and immune response. Scatter graphs of **(A–E)** ubiquitous proinflammatory cytokines, **(F–I)** myeloid inflammatory cytokines, and **(J–S)** lymphoid inflammatory cytokines. Individual values (open circle), mean (colored rectangle), and standard deviation are presented on the graphs. *p < 0.05 between septic shock and control patients. ^#^*p* < 0.05 between COVID-19 and control patients. ^$^*p* < 0.05 between septic shock and COVID-19 patients. CD40L, CD40 ligand; CRP, C-reactive protein; IFNγ, interferon gamma; IL, interleukin; IL-1ra, IL-1 receptor antagonist; IP-10, interferon gamma-induced protein 10; MCP-1, monocyte chemoattractant protein 1; MIP-1α, Macrophage inflammatory protein-1α; sTREM-1, soluble triggering receptor expressed on myeloid cells 1.

The inflammation and immune response substantially differed between COVID-19 and septic shock cases. The CRP elevation and peak were similar in both groups (Figure 4A; Table 1). The septic shock group exhibited a higher neutrophil count, whereas the lymphocyte count at inclusion or nadir during follow-up was similar in both groups (Table 1). Ubiquitous proinflammatory cytokines like TNF-α, IL-1β, IL-6, and IL-8 were discrepantly increased in both groups (Figures 4B–E). Septic shock was characterized by a higher level of myeloid-derived cytokines including IL-1 receptor antagonists (IL-1ra), monocyte chemoattractant protein 1 (MCP-1), macrophage inflammatory protein-1α (MIP-1α), and the soluble form of TREM-1 (Figures 4F–I). In contrast, COVID-19 was distinguished by a lymphocyte T cytokine response (40), as reflected by an increase in IL-2, IL-4, IL-5, IL-7, IL-13, IL-17, and soluble CD40L (sCD40L) (Figures 4J–P). Of note, IFN-γ, IL-10, and IP-10, although elevated, did not differ between COVID-19 and sepsis (Figures 4Q–S).

### Exploratory Multivariate Analysis

**Figure 5:**
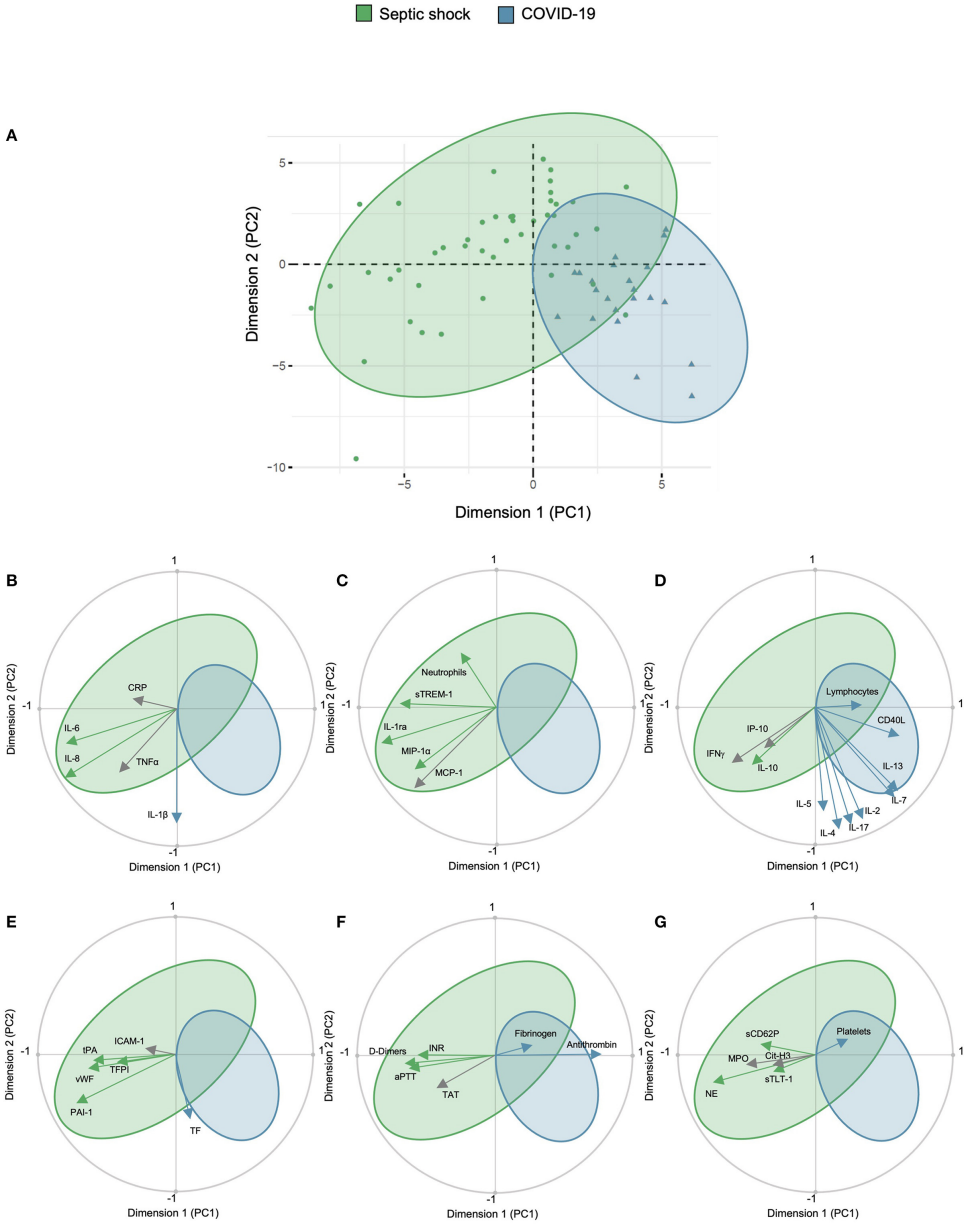
Principal component analysis of study cohort. Principal component analysis showing **(A)** representation of COVID-19 and septic shock population in the two dimensions (score plot). Variables are separated in biplots of **(B)** ubiquitous, **(C)** myeloid, **(D)** lymphoid biomarkers, **(E)** endothelial activation, **(F)** coagulation activation, and **(G)** platelet and NETosis activation with correlation. Scores and loadings are presented in a scatterplot of one principal component (PC) against another. The loadings are represented in a circle of correlations: the closer the arrow of a loading is to the circle, the more the variable is well-represented in the space of the two plotted PCs and contributed to the building of these PCs. This graph also indicates the positive or negative links between the variables and groupings of variables. Arrow colors correspond to the between-group comparison as mentioned in the scatter graphs. CD40L, CD40 ligand; 1Cit-H3, citrullinated histone H3; CRP, C-reactive protein; ICAM-1, intercellular adhesion molecule-1; IFNγ, interferon gamma; IL, interleukin; IL-1ra, IL-1 receptor antagonist; INR, international normalized ration; IP-10, interferon gamma-induced protein 10; MCP-1, monocyte chemoattractant protein 1; MIP-1α, macrophage inflammatory protein-1α; PCA, principal component analysis; MPO, myeloperoxidase; NE, neutrophil elastase; PAI-1, plasminogen activator inhibitor-1; TAT, thrombin–antithrombin complex; TF, tissue factor; TFPI, tissue factor pathway inhibitor; sTLT-1, soluble TREM like transcript-1; tPA, tissue plasminogen activator; sTREM-1, soluble triggering receptor expressed on myeloid cells 1; TT, thrombin time; VCAM-1, vascular cell adhesion protein 1; vWF, von Willebrand factor; WBC, white blood cells.

A PCA was done on the basis of 53 variables to assess the main relations between study outcomes (correlations), patients (similarities among patients from the same group), and between the outcomes and patients. The first two PCs of the PCA captured 39.65% of the total data variability and inflammatory and immune response biomarkers as well as SOFA, PAI-1, NE, and antithrombin ranked among their main contributors (Supplementary Figures 1A–C). The first two PCs allowed the discrimination of COVID-19 and septic shock patients (Figure 5A), consolidating results of the univariate analysis. COVID-19 patients were characterized by an increased IL1β, increased lymphoid activation biomarkers, increased TF, and higher fibrinogen, antithrombin, and platelet count. Septic shock patients were characterized by an increased IL-6 and IL-8, increased myeloid activation biomarkers, endothelial activation biomarkers (except TF), a prolonged coagulation, increased D-Dimers, and platelets activation biomarkers (Figures 5B–G).

## Discussion

For the first time, the inflammation-related coagulopathy of critical COVID-19 and septic shock was prospectively and extensively compared. This study overcame two major limitations of the literature, given that bacterial coinfections were formally excluded from the COVID-19 cohort, while pathological changes were assessed using a control group matched for age, gender, and comorbidities instead of healthy volunteers.

This study highlighted essential clinical differences between critical COVID-19 and septic shock cases. The patients with COVID-19 displayed a longer time delay between symptom onset and ICU admission, and a much longer ICU length of stay, reflecting an extended disease course. Critical COVID-19 patients, in the absence of any bacterial coinfection, rarely presented with hypotension or organ failure. These results contrast with previous observational studies that did not exclude coinfection, demonstrating a higher rate of vasopressor use (41, 42).

Inflammation-induced coagulopathy remarkably differed between COVID-19 and septic shock patients. For instance, COVID-19 was characterized by high circulating TF levels, no platelet or fibrinogen consumption, and lower levels of PAI-1, tPA, and D-dimers, suggesting less fibrinolysis activation. Although TF is recognized as one of the main actors in the inflammatory response during sepsis (43), an increase in circulating TF has never been described outside the DIC context (44). However, an increase in extracellular vesicle TF activity has been shown in COVID-19 (13, 45) and moreover, other viral diseases including human dengue infection and HIV are characterized by an increased soluble TF release (46, 47). Altogether, these results indicate that coagulation abnormalities of COVID-19-associated coagulopathy promote a procoagulant state. In contrast, SIC and DIC are septic shock features, in which the coagulation imbalance can be either pro- or anti-coagulant (48). Therefore, the current study formally supports that COVID-19-associated coagulopathy should not be assimilated to SIC or DIC (49). Given the prolonged disease course, a procoagulant state may explain the high rate of thrombotic complications observed in COVID-19 patients (3, 4).

The histopathological data on COVID-19- and septic-shock-induced ARDS confirmed the presence of lung microthrombi in both diseases. These microthrombi contain numerous Cit-H3^+^-NE^+^ neutrophils at early stages of NET formation, as reported by others (37–39). Neutrophil extracellular traps represent a mechanism by which neutrophils participate to thrombus formation in COVID-19 (19), possibly leading to organ dysfunction (50) and lung injury (51). The similar level of NETosis formation in ARDS due to COVID-19 vs. septic shock suggests that this parameter could be equally involved in the pathophysiology of both diseases. This hypothesis was reinforced by the plasmatic measurements in this clinical cohort revealing identical levels of circulating Cit-H3.

The secretion of soluble markers of platelet activation, namely CD62P and TLT1, was increased in septic shock but unaltered in COVID-19 cases, thereby corroborating previous findings (17). However, this observation remains controversial in the literature (52, 53). The discrepancies could be explained by a small sample size or the possible presence of bacterial coinfections in the critical COVID-19 cohort. Nevertheless, platelets from COVID-19 patients have been reported to display a hyperactive phenotype upon agonist stimulation (8, 17) and it must be noted that soluble CD62P does not inform on the presence of the protein on platelet surface neither on platelet reactivity.

The immune response was characterized by lymphocyte T activation in critical COVID-19 cases, whereas those with septic shock exhibited a greater activation of myeloid cells. Variables related to the immune and inflammatory responses were the most discriminating in distinguishing COVID-19 from septic shock at ICU admission. It has already been shown for patients infected with SARS-CoV-1 that elevation of T helper Type 2 (Th2) cytokines (IL-4, IL-5, IL-10) is correlated with disease severity (54). Interestingly, here, sCD40L also differentiated critical COVID-19 from septic shock patients. sCD40L is expressed by cells of the immune system, especially by activated CD4^+^ T lymphocytes and activated platelets (55). In COVID-19, the elevation of sCD40L was dissociated from the raise of the other platelet activation biomarkers sCD62P and sTLT1, suggesting that its source could be the T lymphocytes. A similar result was recently found in another study (56) and corroborates the elevation of other lymphocyte T-derived ILs. Finally, sCD40L–CD40 interaction promoted endothelial cell and monocyte TF expression (57, 58), linking these two COVID-19 specific observations.

The study had limitations. First, the COVID-19 group was relatively small, so the results must probably be validated in a larger confirmation cohort. However, prospective and systematic enrollment of patients with either COVID-19 or septic shock within a closed time period has limited inclusion bias. Second, the patients with COVID-19 were included during the first wave of the pandemic, when the systematic use of corticosteroids was not yet recommended. It is therefore possible that the study findings would differ in patients that were systematically treated with dexamethasone. Finally, the observations were based on a single time point, namely early after ICU admission. A longitudinal assessment of these specific biomarkers could better define the dynamic changes in inflammatory and coagulation processes over time, which remain to be clarified, particularly in the COVID-19 context.

In conclusion, coagulopathy and inflammation patterns of critical COVID-19 substantially differ from septic shock at ICU admission. Critical COVID-19 is distinguished by very high levels of IL-1β and T lymphocyte activation (including IL-7), whereas septic shock displays higher levels of IL-6, IL-8 and a more significant myeloid response (including TREM-1 and IL-1ra). Moreover, COVID-19-associated coagulopathy is formally different from SIC and DIC, with a marked increase in soluble TF, yet less platelet, antithrombin, and fibrinogen consumption, and less fibrinolysis alteration. Hence, coagulation imbalance of severe COVID-19 almost exclusively tends toward a procoagulant state, and in the context of a prolonged disease course, this could explain the very high rate of thrombo-embolic complications of the disease. A better understanding of critical COVID-19 pathophysiology suggests potential therapeutic strategies, in particular recombinant IL-1ra and recombinant TFPI could modulate these two over-expressed pathways.

## Data Availability Statement

The raw data supporting the conclusions of this article will be made available by the authors, without undue reservation.

## Ethics Statement

The studies involving human participants were reviewed and approved by Comité d'Ethique Hospitalo-Facultaire Saint-Luc-UCLouvain. The patients/participants provided their written informed consent to participate in this study.

## Author Contributions

SH and CBe: had full access to all of the study data and takes responsibility for the data integrity and the accuracy of data analysis. MDec, DC-Z, P-FL, SH, and CBe: concept and design. CBo, AD, SH, LGe, MDec, JDP, MO, LP, VR, AG, JB, DG, M-AVD, JD, HH, LM, MDer, and LJ: acquisition, analysis, or interpretation of data. MDec, JDP, P-FL, SH, and CBe: drafting of the manuscript. VM, LD, and LB: critical revision of the manuscript for important intellectual content. MDec, JDP, MM, and LGa: statistical analysis. MDec, P-FL, LB, SH, and CBe: obtained funding. P-FL and CBe: administrative, technical, or material support. P-FL, SH, and CBe: supervision. MDec, JDP, P-FL, AC, and VM: other—monitoring of the study progress, supporting patient recruitment, data clarifications, and data entry. All authors contributed to the article and approved the submitted version.

## Funding

This work was supported by grants from the *Fondation Saint-Luc* (Brussels, Belgium). The Division of Cardiology at Cliniques Universitaires Saint-Luc, Belgium, has received unrestricted research grants from AstraZeneca (Belgium). MDec is Clinical Master Specialist Applicant to a Ph.D. at the *Fonds National de la Recherche Scientifique et Médicale* (FNRS, Belgium). JDP was supported by a grant from the Salus Sanguinis Foundation (UCLouvain, Belgium). MO, LP, and JB are supported by Fund for Research Training in Industry and Agriculture (FRIA, FNRS). VM is post-doctorate Clinical Master Specialist at the FNRS. LD and SH are senior research associates at FNRS. QUALIblood s.a. offered the analyses for determining the cytokinic profile.

## Conflict of Interest

JD is the CEO and founder of QUALIblood s.a., a Belgian Contract Research Organization. LM and HH were employed by the company QUALIblood s.a. and authors MDer and LJ were employed by the company Inotrem s.a. The remaining authors declare that the research was conducted in the absence of any commercial or financial relationships that could be construed as a potential conflict of interest. The authors declare that this study received funding from Foundation Saint-Luc. The funder was not involved in the study design, collection, analysis, interpretation of data, the writing of this article or the decision to submit it for publication.

## Publisher's Note

All claims expressed in this article are solely those of the authors and do not necessarily represent those of their affiliated organizations, or those of the publisher, the editors and the reviewers. Any product that may be evaluated in this article, or claim that may be made by its manufacturer, is not guaranteed or endorsed by the publisher.
