# Supplementary material for: Inflammation-Induced Coagulopathy Substantially Differs Between COVID-19 and Septic Shock: A Prospective Observational Study
Source: Front Med (Lausanne). 2022 Jan 17;8:780750. doi: 10.3389/fmed.2021.780750 (PMC8801505; doi:10.3389/fmed.2021.780750)
Supplement: Supplementary file 1 [file Data_Sheet_1.PDF]

Supplemental material.

Supplemental methods: Lung histopathology

Histopathological characterization of lungs and multiplex immunostaining (mIS)

Lung samples (from biopsies or autopsies) were fixed in 4% formaldehyde, embedded in paraffin and sectioned. After deparaffinization, 5µm tissue sections were processed according to the protocol described by Aboubakar et al. (1). Endogenous peroxidases were inhibited by means of a 20-min treatment with 3% hydrogen peroxide in methanol. Sections were then blocked in tris buffered saline (TBS) supplemented with 5% bovine serum albumin (BSA) and 0.1% Tween 20. The primary antibody was incubated for 1 hour at room temperature or overnight at 4°C (anti-CD31) in TBS containing 1% BSA and 0.1% Tween 20, then detected using corresponding horseradish peroxidase (HRP)-conjugated polymer secondary antibody incubated for 40-min at room temperature. HRP was then visualized by tyramide signal amplification (TSA) using AlexaFluor-conjugated tyramides. After antigen retrieval in 10mM citrate buffer pH 5.7 (this step also removes antibodies from tissue sections), the same protocol was applied using other primary antibodies and AlexaFluor or Atto-conjugated tyramides. In this study, four sequential incubations were performed, as indicated in ESM Table 2. After a washing in phosphate buffer saline, nuclei were stained with Hoechst 33342 (Thermo Fisher Scientific) diluted in TBS containing 10% BSA and 0.1% Tween 20, and sections were mounted in Dako fluorescence mounting medium (Agilent). Slides were stored at 4°C until multispectral whole slide imaging at X20 magnification with an Axioscan.z1 slide scanner (Zeiss). All reagents and antibodies are listed in the following table.

Sequence	Antigen	Primary antibody			Secondary antibody		TSA		
		Dilution	Company	Catalog number	Polymer HRP	Company	Company	Catalog number	Dye
1	NE	1/250	Millipore	481001	Envision anti-rabbit	Agilent (Dako)	Thermo	B40955	AF555
2	CD31	1/2000	Abcam	AB212709	Envision anti-mouse	Agilent (Dako)	Thermo	B40953	AF488
3	Cit-H3	1/5000	Abcam	Ab5103	Envision anti-rabbit	Agilent (Dako)	Thermo	B40958	AF647
4	CD42b	1/500	Invitrogen	MA5-11642	Envision anti-mouse	Agilent (Dako)	Home-made		Atto425

Abbreviation: NE, neutrophil elastase; Cit-H3, citrullinated histone H3; HRP, horseradish peroxidase; TSA, tyramide signal amplification

Computer-assisted quantitative evaluation of immunostaining in whole tissue sections

Immunostainings were quantified on multiplex-stained paraffin sections of standardized representative tissue sections using the image analysis tool Author Version 2017.2 (Visiopharm, Hørsholm, Denmark). Tissue sections were first automatically delineated at low digital magnification based on the nuclei and 594 staining. Within the delineated tissue, vascular structures were automatically detected based on CD31 staining. Manual correction was applied to exclude artefacts and further refine macrovessel detection. Intra-vascular Cit-H3^{+}-NE^{+} neutrophils and platelets were then detected with a thresholding classification method based on AF555, AF647 and Atto425 staining, respectively. The same parameters

were kept constant for all slides. Results were expressed as the part per million of stained vessel area (neutrophil elastase, Cit-H3, or CD42b area above threshold $*10^6$/analyzed area).

Reference

1. Aboubakar Nana F, Hoton D, Ambroise J, Lecocq M, Vanderputten M, Sibille Y, et al. Increased Expression and Activation of FAK in Small-Cell Lung Cancer Compared to Non-Small-Cell Lung Cancer. Cancers (Basel). 2019;11(10).

Supplemental Table 1: Reagents used for plasma biomarkers measurements

Biomarker	Company	Catalog number
TF	R&D systems	#DCF300
TFPI	R&D systems	#DTFP10
vWF	HemosIL	#0020002300
PAI-1	T coag Stago TriniLIZE	#T6003
tPA	R&D systems	#DTPA00
ICAM-1	Bio-Rad	#171B6009M
VCAM-1	Bio-Rad	#171B6009M
TAT	R&D systems	#OWMG15
Antithrombin	HemosIL	#0020030100
sCD62P	R&D systems	#DPSE00
sTLT-1	R&D systems	#DY2394 + #DY008
MPO	R&D systems	#DMYE00B
Cit-H3	Cayman	#501620
NE	R&D systems	#DY9167 + #DY008
IFNγ	Bio-Rad	#M500KCAF0Y
IL-1β	Bio-Rad	#M500KCAF0Y
IL-1ra	Bio-Rad	#M500KCAF0Y
IL-2	Bio-Rad	#M500KCAF0Y
IL-4	Bio-Rad	#M500KCAF0Y
IL-5	Bio-Rad	#M500KCAF0Y
IL-6	Bio-Rad	#M500KCAF0Y
IL-7	Bio-Rad	#M500KCAF0Y
IL-8	Bio-Rad	#M500KCAF0Y
IL-9	Bio-Rad	#M500KCAF0Y
IL-10	Bio-Rad	#M500KCAF0Y
IL-13	Bio-Rad	#M500KCAF0Y
IL-17	Bio-Rad	#M500KCAF0Y
IP-10	Bio-Rad	#M500KCAF0Y
MCP-1	Bio-Rad	#M500KCAF0Y
MIP-1α	Bio-Rad	#M500KCAF0Y
sCD40L	R&D systems	#DCDL40
sTREM-1	R&D systems	#DTRM10C

Abbreviation: TF, tissue factor; TFPI, tissue factor pathway inhibitor; vWF, von Willebrand factor; PAI-1, plasminogen activator inhibitor-1; tPA, tissue plasminogen activator; ICAM-1, intercellular adhesion molecule-1; TATc, thrombin antithrombin complex; ATIII, antithrombin III; sTLT-1, soluble TREM like transcript-1; MPO, myeloperoxidase; Cit-H3, citrullinated histone H3; NE, neutrophil elastase; IFNγ, interferon gamma; IL, interleukin; IL-1ra, IL-1 receptor antagonist; IP-10, IFNγ-induced protein 10; MCP-1, monocyte chemoattractant protein-1; MIP-1α, macrophage inflammatory protein 1α; sTREM-1, soluble triggering receptor expressed on myeloid cells 1

Supplemental Table 2: Type of infection and micro-organisms in septic shock patients

Culture	N = 48
**Gram-negative organisms**	**31 (64.1)**
Escherichia coli	13 (27.1)
Klebsiella	3 (6.3)
Pseudomonas aeruginosa	4 (8.4)
Enterobacter spp	4 (8.4)
Haemophilus influenzae	1 (2.1)
Other Gram-negative bacilli	6 (12.5)
**Gram-positive organisms**	**13 (27.1)**
Staphylococcus aureus	5 (10.4)
Streptococcus pneumonia	6 (12.5)
Group A streptococcus spp	2 (4.2)
**Anaerobes**	**1 (2.1)**
**Other organisms**	**3 (6.3)**
**Site of infection**	**N=48**
Pneumonia	10 (20.8)
Intraabdominal	15 (31.2)
Skin and soft tissue	4 (8.4)
Genitourinary	9 (18.7)
Intravascular catheter related	2 (4.2)
Primary bloodstream infection	3 (6.3)
Other	5 (10.4)

Values are numbers (percentages)

Supplemental Table 3: Baseline characteristics of patients in histopathological analysis and clinical statistics.

	**Control** n=4	**COVID-19** n=6	**Septic Shock** n=4	p value
**Demographics**				
Men	3 (75)	3 (50)	3 (75)	0.63
Women	1 (25)	3 (50)	1 (25)	
Age (years)	50 ± 21	67 ± 16	56 ± 28	0.51
**Medical history**				
Hypertension	1 (25)	3 (50)	1 (25)	0.63
BMI > 25	2 (50)	4 (67)	3 (75)	0.75
Diabetes	0 (0)	1 (17)	0 (0)	0.49
History of smoking	2 (50)	1 (17)	1 (25)	0.51
COPD	0 (0)	1 (17)	0 (0)	0.49
Cancer	6 (100)	2 (33)	1 (25)	0.02
**Sampling method**	Biopsy	Autopsy	Biopsy	
**Organ failure and severity scores**				
PaO_2/FiO_2		99 ± 32	176 ± 85	0.07
Apache II score		22 ± 7	20 ± 12	0.46
SOFA Score		9 ± 3	7 ± 5	0.26

Values are numbers (percentages) or mean ± standard deviation.

Abbreviations: BMI, body mass index; COPD, chronic obstructive pulmonary disease; PaO_2/FiO_2, arterial oxygen partial pressure/fractional inspired oxygen; SOFA, sepsis-related organ failure assessment.

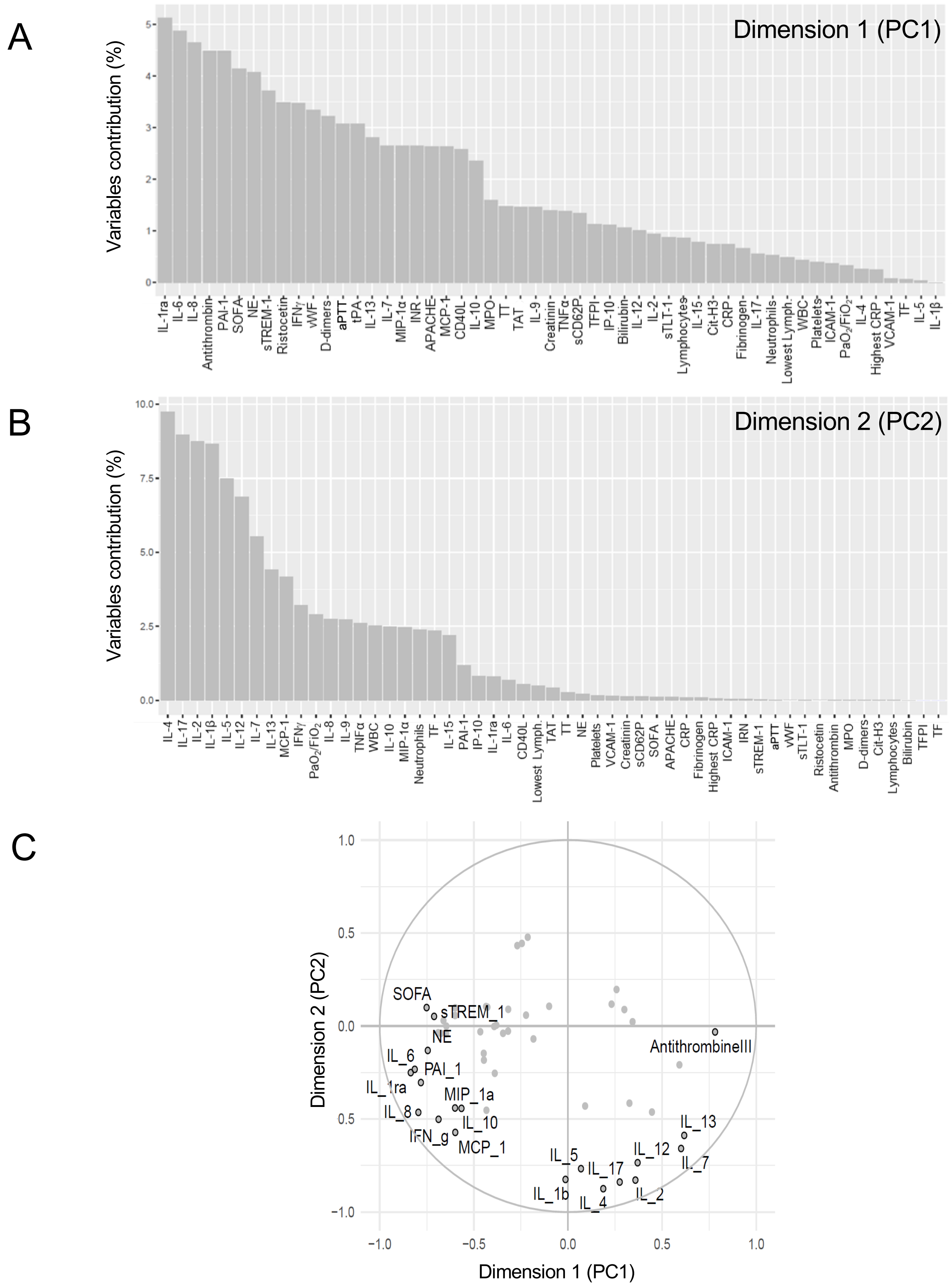

Supplemental Figure S1: Principal component analysis of study cohort

Contribution of variables in establishment of dimensions of (A) principal component (PC) 1 and (B) 2. (C) Representation of COVID-19 and septic shock population in the two dimensions (scores plot) and the variables that are best represented in this plane ($cos^2 \geq 0.5$). Abbreviation: IFNγ, interferon gamma; IL, interleukin; IL-1ra, IL-1 receptor antagonist; MCP-1, monocyte chemoattractant protein-1; MIP-1α, macrophage inflammatory protein 1α; NE, neutrophil elastase; sTREM-1, soluble triggering receptor expressed on myeloid cells 1.
